# Yeast-Based Fluorescent Sensors for the Simultaneous Detection of Estrogenic and Androgenic Compounds, Coupled with High-Performance Thin Layer Chromatography

**DOI:** 10.3390/bios10110169

**Published:** 2020-11-08

**Authors:** Liat Moscovici, Carolin Riegraf, Nidaa Abu-Rmailah, Hadas Atias, Dror Shakibai, Sebastian Buchinger, Georg Reifferscheid, Shimshon Belkin

**Affiliations:** 1 Department of Plant and Environmental Sciences, Institute of Life Sciences, Hebrew University of Jerusalem, Jerusalem 91904, Israel; liatmosc@savion.huji.ac.il (L.M.); nidaan8@gmail.com (N.A.-R.); hadas.atias@mail.huji.ac.il (H.A.); drors1987@gmail.com (D.S.); 2 Department Biochemistry, Ecotoxicology, Federal Institute of Hydrology (BfG), Am Mainzer Tor 1, 56068 Koblenz, Germany; carolin.riegraf@gmail.com (C.R.); Buchinger@bafg.de (S.B.); reifferscheid@bafg.de (G.R.); 3 RWTH Aachen University, Department of Ecosystem Analysis, Worringerweg 1, D-52074 Aachen, Germany

**Keywords:** Bioassays, high performance thin layer chromatography, endocrine disrupting compounds, fluorescent proteins, wastewater

## Abstract

The persistence of endocrine disrupting compounds (EDCs) throughout wastewater treatment processes poses a significant health threat to humans and to the environment. The analysis of EDCs in wastewater remains a challenge for several reasons, including (a) the multitude of bioactive but partially unknown compounds, (b) the complexity of the wastewater matrix, and (c) the required analytical sensitivity. By coupling biological assays with high-performance thin-layer chromatography (HPTLC), different samples can be screened simultaneously, highlighting their active components; these may then be identified by chemical analysis. To allow the multiparallel detection of diverse endocrine disruption activities, we have constructed *Saccharomyces cerevisiae*-based bioreporter strains, responding to compounds with either estrogenic or androgenic activity, by the expression of green (EGFP), red (mRuby), or blue (mTagBFP2) fluorescent proteins. We demonstrate the analytical potential inherent in combining chromatographic compound separation with a direct fluorescent signal detection of EDC activities. The applicability of the system is further demonstrated by separating influent samples of wastewater treatment plants, and simultaneously quantifying estrogenic and androgenic activities of their components. The combination of a chemical separation technique with an optical yeast-based bioassay presents a potentially valuable addition to our arsenal of environmental pollution monitoring tools.

## 1. Introduction

Endocrine disrupting chemicals (EDCs) are exogenous agents with structural similarity to endogenous hormones, that may therefore interfere with natural hormonal activity by blocking, competing or mimicking natural hormones [1]. The biological effects associated with exposure to EDCs include numerous physiological processes, among them homeostasis disruption, immunological damages and developmental impairments [2]. Furthermore, some EDCs were suggested to act as carcinogenic agents [3]. These adverse health effects, in some cases triggered by exposure to parts-per-billion level concentrations [4,5,6], have raised concern among public health authorities. This concern is exacerbated when considering the possible manifestation of certain health effects associated with exposure to EDCs across generations [2]. Besides being a human health risk, the release of EDCs to aquatic systems also poses a significant ecological threat. Continuous exposure to such chemicals may affect local species and ecosystems. It has been shown, for example, that chronic exposure to EDCs causes abnormalities in the reproductive system of certain fish species [1,7,8,9].

The estrogen (ER) and the androgen (AR) receptors are prominent members of a hormone receptor superfamily that mediates a wide range of significant biological activities. These vary from reproductive development to the regulation of the cardiovascular system, the immune system, the central nervous system, and more [10,11]. Both of these steroid hormone receptors are comprised of three main structural domains with similar functionalities: (a) the N-terminal transcription regulation domain; (b) the ligand binding domain (LBD), which attaches to the target ligand, prompting a conformational change that allows the receptor–ligand complex to enter the nucleus; and (c) the DNA binding domain. A ligand–receptor dimer complex is translocated into the nucleus, and binds to a DNA consensus sequence at the promoter of a target gene, known as the Hormone Response Element (HRE), leading to the transcription of the gene [2,10,12].

Detecting specific pollutants that exhibit hormonal activity in complex environmental samples, e.g., treated wastewater, is very challenging due to the complexity of the matrix. Such samples may contain a large variety of EDCs, as well as numerous unknown EDC metabolites, which may also exert endocrine disrupting activity. The need to focus on the presence of unknown but bioactive compounds, renders traditional detection methodologies, e.g., liquid- or gas-chromatography coupled to mass spectrometry (LC/MS and GC/MS, respectively), less suitable for the detection of EDCs in complex samples [12,13].

A possible alternative to such methods is the use of whole-cell biosensors, genetically engineered to emit a detectable signal upon exposure to chemicals exerting hormonal activity. Such effect-based methods require no prior information regarding the chemical structure of the EDCs in the sample. When applied in a microtiter plate-based assay, this methodology allows quantifying the combined biological effect resulting from exposure to the EDCs in the sample, rather than assessing the concentrations of individual chemicals. This feature adds important information on the toxic effects of compound mixtures. A notable disadvantage of this approach is the inability to distinguish between different components exerting similar biological effects [14]. To overcome this difficulty, a number of reports describing the direct coupling of effect-based methods to high-performance thin-layer chromatography (HPTLC) have been published recently [13,15,16]

The combination of chemical separation by high-performance thin-layer chromatography (HPTLC) and the effect-based assay by yeast-based sensor strains allowed the separation of environmental samples and the discovery of individual sample components exhibiting hormonal activity. These active compounds can then be removed from the HPTLC plate and identified via traditional analytical methods (e.g., LC/MS, GC/MS) [17,18]. However, since a separate assay has to be conducted for each biological endpoint, the throughput potential of this approach is low. In an answer to this need, the present article describes the development of yeast (*Saccharomyces cerevisiae*)-based sensors that detect the presence of chemicals exerting androgenic and estrogenic activity by expressing spectrally different fluorescent proteins. Following characterization of the constructed sensor strains in a 96-well microtiter plate format, they were sprayed over HPTLC plates, in which model compounds and later wastewater samples were separated. Following incubation, EDCs with estrogenic and androgenic activities were simultaneously detected in the same sample. In contrast to a previously described *Arxula adeninivorans*-based assay with similar objectives [19], we have employed a spray-on-technology to apply a uniform layer of the yeast bioreporters to the HPTLC surface [20]. This methodology allows the control of the thickness of the suspension layer and produces clear and sharp bands, as opposed to an immersion procedure.

## 2. Materials and Methods

### 2.1. Chemicals

Testosterone (CAS: 58-22-0) and 5α-androstan-17β-ol-3-one (DHT, CAS: 521-18-6) were used as androgenic reference compounds. Estrone (E1, CAS: 53-16-7), 17β-estradiol (E2, CAS: 50-28-2), estriol (E3, CAS: 50-27-1), and 17α-ethinylestradiol (EE2, CAS: 57-63-6), were used as estrogenic reference compounds. These chemicals were of the highest analytical grade and were purchased from Sigma-Aldrich. Stock solutions of reference compounds (0.5 mg/mL for DHT, 5 mg/mL for the rest) were prepared in ethanol. Chromatographic separation was performed on silica gel HPTLC plates of type 60G F_254_ (20 × 10 cm or 10x10 cm) purchased from Merck. Solvents used for HPTLC were of the highest analytical grade and were purchased from Merck.

### 2.2. Yeast Strains, Plasmids, and Growth Conditions

Two previously constructed *S. cerevisiae* sensor strains and two plasmids were employed in this study as a basis for the construction of the new fluorescent bioreporters. The two strains, harboring either the human estrogen nuclear receptor (hER) or the human androgen receptor (hAR), integrated into the yeast genome, were purchased from BioTech (Knoxville, TN, USA). The two plasmids were kindly donated by Prof. S. Ripp (University of Tennessee, Knoxville, TN, USA). Plasmids pUTK407 and pUTK420 [21,22], contained an estrogenic or an androgenic hormone response element (HRE), respectively, between bidirectional constitutive and strong yeast promoters, upstream of the *luxA* and *luxB* genes of the luminescent bacterium *Photorhabdus luminescens*. Plasmid pUTK407 carries two copies of the human estrogen HRE, located between the constitutive divergent promoters GPD and ADH1. Similarly designed, pUTK420 carries four copies of the human androgen HRE, located between the same two promoters. The *luxA* and *luxB* genes are located downstream of the GPD and ADH1 promoters, respectively. The palindromic nature of the HRE region forms a hairpin structure that represses the activation of the GPD and ADH1 promoters. Upon binding of the ligand–receptor complex to its respective HRE, this hairpin structure is released, and both *luxA* and *luxB* are divergently transcribed, yielding the two structural subunits of the bacterial luciferase.

**Figure 1:**
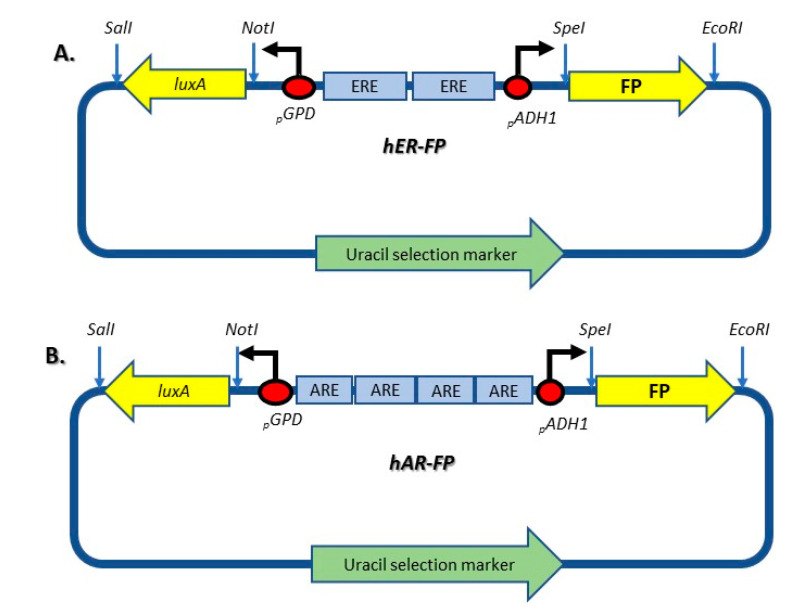
Schematic design of the hER-FP (**A**) and hAR-FP (**B**) plasmids used in this study for the detection of estrogenic and androgenic activity, respectively. Plasmid hER-FP (**A**), derived from plasmid pUTK407 (21), contains two copies of the human estrogen response element (ERE). Plasmid hAR-FP (**B**), derived from plasmid pUTK420 (22), contains four copies of the human androgen response element (ARE). Upon binding of a receptor–ligand complex to its respective response element, a hairpin structure is released and activation of the constitutive GPD and ADH1 promoter is enabled, resulting in transcription of *luxA* and fluorescent genes (FP), either EGFP, BFP, or Ruby.

In the present study, the *luxB* sequences from the skeleton plasmids [21,22] were replaced by one of three fluorescent protein (FP) genes (Figure 1): green (EGFP), red (mRuby2), or blue (mTagBFP2). These genes were extracted from plasmids pFA6a-link-yoEGFP-spHIS5, pFA6a-link-yomRuby2-spHIS5 and pFA6a-link-yomTag BFP2-spHIS5, respectively [23]. All three plasmids were a kind gift from Wendell Lim and Kurt Thorn (Addgene plasmids #44,838, #44,858 and #44,836, respectively). A set of complementary oligonucleotides, for each of the fluorescent protein sequences, was obtained using KOD Hot Start DNA polymerase (Merck). The six new plasmids generated in this manner are listed in Table 1, and the PCR primers employed for cloning the fluorescent protein genes are listed in Table S1. DNA manipulations were performed according to standard protocols [24].

Yeast extract–peptone–dextrose (YPD) liquid medium was used for routine growth of the plasmid-free *S. cerevisiae* strains. A modified minimal medium without uracil (Sigma-Aldrich/Formedium, United Kingdom) was employed to grow strains harboring plasmids with a uracil selective marker.

Transformation of plasmids into *S. cerevisiae* cells was performed according to a standard lithium acetate protocol [27]. Briefly, *S. cerevisiae* cells were grown overnight (30 °C, 200 rpm), and then diluted 100-fold into 10 mL of fresh medium. The cells were grown under the same conditions to late exponential growth phase, until the optical density at 600 nm (OD_600_) was 0.6 to 1, and then washed with nuclease-free water, and pelleted at 10,600 RCF (Eppendorf 5417C) at room temperature. Following a second washing step with 0.1 M lithium acetate (CAS: 6108-17-4, Sigma-Aldrich), the cells were resuspended in 240 μL 50% polyethylene glycol (PEG 4000, CAS: 25322-68-3, Merck). Subsequently, 36 μL of 1 M lithium acetate, 25 μL of carrier DNA (10 mg/mL, fish testes denatured DNA, CAS: 100403-24-5, USA Bioworld) and 45 μL of the DNA to be transformed (up to 1 μg) were added. The cells were then incubated at 30 °C on a gently rotating platform for 45 min, following which they were subjected to a 25 min heat shock at 42 °C. The cells were pelleted, resuspended in 50 μL of nuclease-free water, and plated on minus *ura* synthetic complete (SC) agar plates [28]. Successful transformations were verified by colony PCR and sequencing.

### 2.3. Endocrine Assay in 96-Well Plates

Yeast strains were grown overnight (30 °C, 250 rpm) in a selective medium (a synthetic complete medium, lacking uracil, unless mentioned otherwise). The culture was diluted 100-fold in fresh medium, and re-grown under the same conditions to late exponential growth phase (O.D.600 = 0.6–1). Aliquots (40 μL) of the culture were then dispensed into each well of a 96-well black clear-bottom microtiter plate (Greiner), containing 80 µL of reference compounds at predetermined concentrations (0.0122–200 µg/l). The reference compounds, either E2 (estradiol), or testosterone, were dissolved in ethanol, which also served as a negative control (1%).

The 96-well plates were incubated at 30 °C for 18 h ± 1 h in a TECAN plate reader (Infinite M200 PRO), and the fluorescent signal was read every hour, following a 10 sec vigorous shaking of the plate. The readings were performed using excitation/emission wavelengths of 559/600 nm for Ruby, 488/507 nm for EGFP and 399/454 nm for BFP. Fluorescence values are displayed as the instrument’s arbitrary relative fluorescence units (RFU).

### 2.4. Calculation of the Corrected Fluorescence and the Reporter Gene Induction in 96-Well Plates

A corrected fluorescence [13] value, accounting for cell density as well as background fluorescence, was calculated according to the following equation:

$$F_{c}\left(i\right)=\frac{\left[A_{Fluorescence}\left(i\right)-\bar{B_{fluorescence}}\left(i\right)\right]}{\left[A_{600}\left(i\right)-\bar{B_{600}}\left(i\right)\right]}$$

where Fc(i) is corrected fluorescence for test i (sample dilutions, reference dilutions, negative control); $A_{fluorescence}\left(i\right)\;$is fluorescence intensity for test i; $\bar{B_{fluorescence}}\left(i\right)\;$is mean fluorescence intensity for blank replicates of test i; $A_{600}\left(i\right)$ is absorbance at 600 nm for test i; and $\bar{B_{600}}\left(i\right)$ is absorbance at 600 nm for blank replicates of test i.

### 2.5. Endocrine Assay on the HPTLC Plate Surface

HPTLC plates (Silica gel, F_254_, 20 × 10 cm, Merck) were developed with methanol to 5 mm below the rim, dried at 120 °C for 30 min, and stored in a desiccator at room temperature until used. Samples and reference compounds were applied by an Automatic TLC Sampler 4 (ATS 4, CAMAG, Muttenz, Switzerland), in amounts ranging from 0.5 pg to 1000 pg per spot, as described before [13]. Samples were focused with 100% methanol to a distance of 20 mm, followed by 5 min drying in a chemical hood. Chromatographic development, up to 10 mm below the rim, was performed using an Automated Multiple Development System (AMD 2, CAMAG, Muttenz, Switzerland). For the separation of estrogen-like compounds, a chloroform/acetone/petroleum ether (55:20:25) mixture was used as the mobile phase. Ethylacetate/n-hexane (50:50) served as the mobile phase for the separation of androgen-like compounds [13,29]. For the simultaneous detection of estrogenicity and androgenicity, a mobile phase consisting of ethylacetate/n-hexane (50:50) was employed. Following separation, the plates were dried in a chemical hood until the organic solvents evaporated [13].

For the detection of endocrine activity on the HPTLC plate, an overnight culture of the yeast-based bioreporters was centrifuged at 10,600 RCF for 5 min (Eppendorf centrifuge 5417C). The pellet was then resuspended in fresh minimal medium without uracil, and regrown under the same conditions to late exponential growth phase (OD_600_ = 0.6–1). The cells were sprayed homogenously on the developed HPTLC plate, either manually with a glass reagent sprayer (CAMAG, Muttenz, Switzerland), or by using an automated spraying device (CAMAG Derivatizer, CAMAG, Muttenz, Switzerland, 2.5 mL, spraying level 3, yellow nozzle). Images of the fluorescent signal were obtained after an incubation of 4 h to 18 h at 30 °C in an opaque plastic box, in which humidity was maintained by a water-soaked paper towel. The fluorescent EGFP signals were detected using Fusion FX imaging system (Vilber Lourmat) at excitation and emission wavelength of 365 nm 565 nm, respectively. The fluorescent Ruby and BFP signals were detected using a TLC Scanner 4 (CAMAG) operated under the *visionCATS* software (version 2.3. SP1, CAMAG, Muttenz, Switzerland). Ruby signals were detected at λ_ex_ = 525 nm with a cutoff filter of 540 nm, and BFP signals at λ_ex_ = 396 nm and a cutoff filter of 400 nm. Additionally, qualitative assessment was performed on images acquired with a TLC Visualizer 2 (CAMAG) operated under the *visionCATS* software (version 2.3. SP1,) under long wavelength UV light (λ _em_ = 366 nm).

### 2.6. Preparation of Wastewater Samples

Freshly collected influent samples of municipal wastewater treatment plants were centrifuged (Thermo Scientific, Sorvall RC 6 Plus Centrifuge, 17,000 RCF, 20 min) and the supernatant was filtered through a glass fiber filter (Pall, type A/C, Ø 47 µm). Filtered samples were concentrated by solid-phase extraction (SPE) using Oasis HLB cartridges (200 mg, 6 mL). The columns were conditioned by the successive application of 2 mL n-heptane, 2 mL acetone, three aliquots of 2 mL methanol, and four aliquots of 2 mL deionized water. Methanol (2 × 4 mL) was used to elute the adsorbed sample components from the cartridges. The extracts were reduced to 500 µL using a Turbo Vap II Concentration Workstation (Biotage AB, Uppsala, Sweden) under a gentle nitrogen flow. The extracts were transferred into amber glass vials. The evaporation tubes were rinsed three times with methanol, which was then used to fill up the extracts to a final volume of 1 mL, resulting in a final 200-fold enrichment. The extracts were stored at −20 °C until use.

### 2.7. Fluorescent Microscopy

Microscope images were obtained using a VF1200 confocal microscope (Olympus, Tokyo, Japan) with a 60 × 1.42 oil objective. The excitation wavelengths and emission filters were 488/507 nm for EGFP, 559/600 nm for Ruby and 399/454 nm for BFP.

### 2.8. Data Processing and Statistical Analysis

Statistical analysis of the HPTLC combined bioassays was performed as previously described [13], using the intensity (in arbitrary units) of the determined peak areas obtained with the *visionCATS* software (version 2.5. SP1, CAMAG). Data were further processed using Excel^®^ and R version 3.5.2 (R Core Team, Vienna, Austria) [30], the ‘drc’ [31] and the ‘ggplot’ [32] packages. Signal-to-noise-ratios (S/N) were determined to calculate the limit of detection (LOD) and limit of quantification (LOQ) with S/N ≥ 3 and S/N ≥ 10, respectively.

## 3. Results

### 3.1. Sensor Strain Characterization in a 96-Well Plate Assay

**Figure 2:**
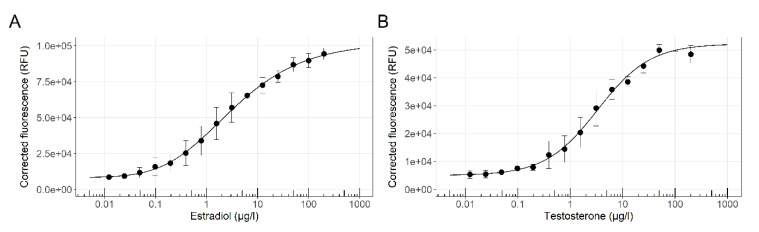
Dose-response curves of two fluorescent bioreporters. (**A**) Response of strain ER-Ruby to β-Estradiol (E2; 0.0122–200 µg/L); (**B**) Response of strain AR-BFP to testosterone (0.0122–200 µg/L). Estrogenic and androgenic activities were determined after an incubation time of 18 h at 30 °C. Corrected fluorescence values were calculated as detailed in Materials and Methods (Section 2.4), and the error bars show the respective standard errors. The solid line was fitted to the data using a five-parameter log-logistic function.

To reach the study’s objective, developing a method for multi parallel detection of different endocrine disruptors in environmental samples within a single assay, we have designed and constructed a battery of yeast-based sensor strains. The six members of the sensor panel, addressing two target chemical groups (with estrogenic or androgenic activities), with three reporter proteins (green, red and blue) each, are listed in Table 1. The responses to their designated model targets have first been characterized in a conventional 96-well microtiter plate procedure in liquid medium. The activities of two bioreporters out of this list, an estrogenic sensor (ER-Ruby) and an androgenic (AR-BFP) are presented, as an example, in Figure 2.

The response of both strains to increasing concentrations of their respective reference compounds followed a classic asymmetrical logistic dose-response curve. Both strains exhibited high sensitivity towards their reference compounds; calculated LOD values for 17β-estradiol (E2) by ER-Ruby and testosterone by AR-BFP were 0.032 and 0.070 µg/L, respectively.

**Figure 3:**
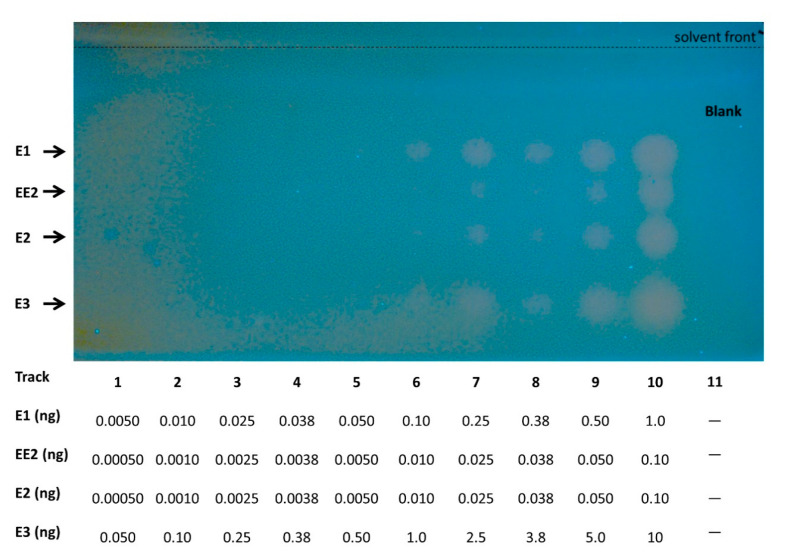
Detection of estrogenic activity by the ER-Ruby sensor strain following high-performance thin-layer chromatography (HPTLC) separation. Different amounts (indicated below the image) of a mixture consisting of the reference compounds estrone (E1), 17α-ethinylestradiol (EE2), 17β-estradiol (E2), and estriol (E3) were separated by a two-step chromatographic development using methanol and a chloroform/ethyl acetate/petroleum ether mixture (55:20:25, *v*/*v*/*v*). Ethanol served as blank on track 11. Following an 18 h incubation at 30 °C, fluorescence was imaged using a TLC Visualizer 2 at λ_ex_ = 366 nm. Signals were enhanced using the enhancement tool of the *visionCATS* software.

**Figure 4:**
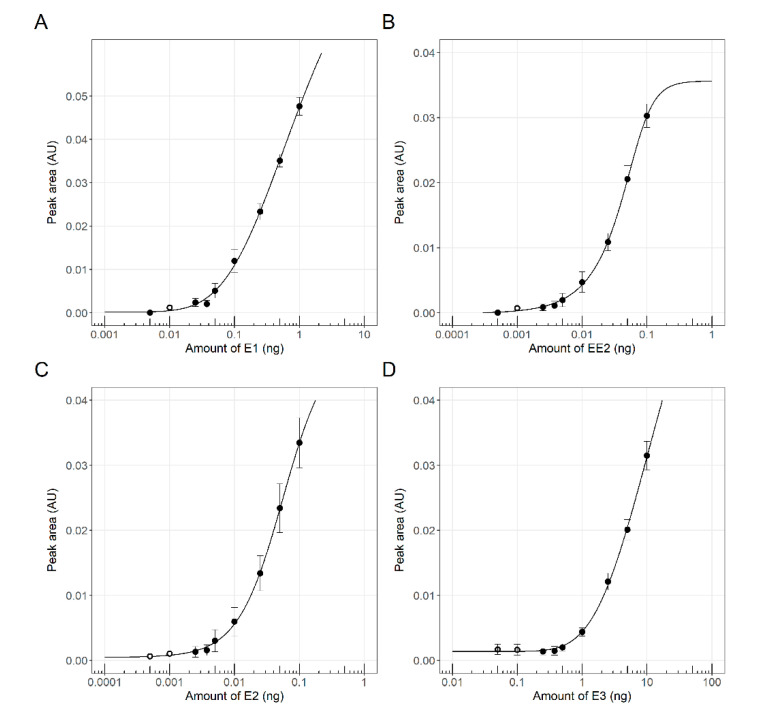
Dose-response curves of estrogenic reference compounds Estrone (E1, **A**), 17α-Ethinylestradiol (EE2, **B**), 17β-Estradiol (E2, **C**), and Estriol (E3, **D**), as derived from the experiment described in Figure 3 above. Fluorescent signal intensity determined by the peak area was plotted against the applied amount. Data points represent the mean values of the signal and the error bars show the respective standard error (n_black_ = 3, n_grey_ = 2 and n_white_ = 1). The solid lines were fitted to the data using a five-parameter log-logistic function.

**Figure 5:**
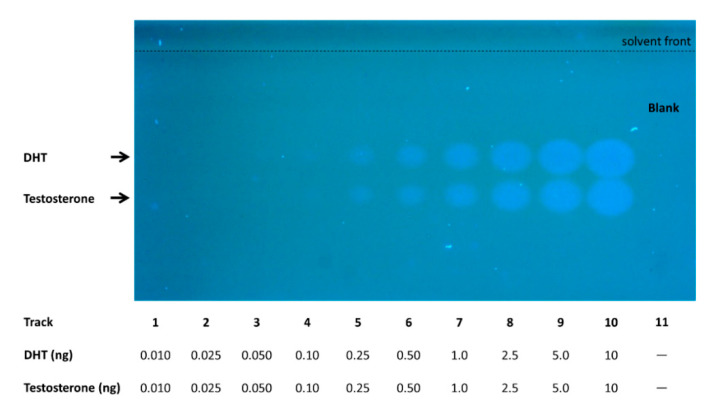
Detection of androgenic activity by the AR-BFP sensor strain following HPTLC separation. Different amounts (indicated below the image) of a testosterone/DHT (5α-androstan-17β-ol-3-one) mixture were separated by a two-step chromatographic development using methanol and an ethyl acetate/n-hexane mixture (50:50, *v*/*v*). Ethanol served as blank on track 11. Following an 18 h incubation at 30 °C, fluorescence was imaged using a TLC Visualizer 2 at λ_ex_ = 366 nm. Signals were enhanced using the enhancement tool of the *visionCATS* software.

**Figure 6:**
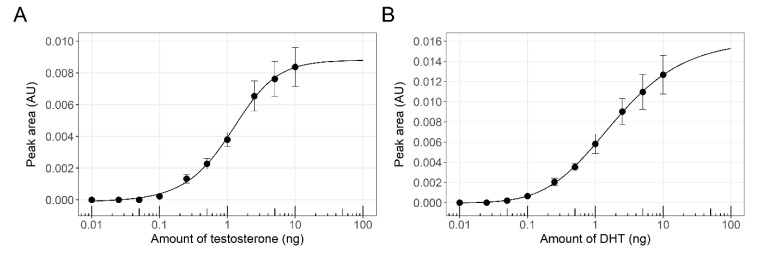
Dose-response curves of androgenic reference compounds testosterone (**A**) and 5α-Androstan-17β-ol-3-one (DHT, **B**), as derived from the experiment described in Figure 5 above. Fluorescent signal intensity determined by the peak area detected using a TLC Scanner 4 (λ_ex_ = 396 nm, cut-off filter of 400 nm) was plotted against the applied amount. Data points represent the mean values of the signal, and the error bars show the respective standard error (n = 3). The solid lines were fitted to the data using a five-parameter log-logistic function.

Thin-layer chromatography allows the separation of compounds based on polarity-governed partitioning between the solid and the mobile phases. A planar yeast estrogen screen (pYES) to investigate estrogenic activities of environmental sample components was previously described, using yeast bioreporters coupled with HPTLC [29,33,34]. Recently, Riegraf et al. [13] demonstrated that additional modes of action can be addressed by combining other yeast-based reporter gene assays with HPTLC, including androgenic effects. In all of these cases, β-galactosidase has been employed as the reporter entity, and only one type of endocrine activity could be assayed in a single plate. In the present study, the optimized working protocols described in these publications have been used to test the performance of the newly developed fluorescent yeast strains. Mixtures of estrogenic [E1 (estrone), E2 (17β- estradiol), EE2 (17α- ethinylestradiol) and E3 (estriol)] and androgenic [DHT (dihydrotestosterone) and testosterone] reference compounds were applied and subsequently separated by HPTLC, as described under Material and Methods. The yeast fluorescent bioreporter cells were then sprayed as a thin layer on top of the silica plate, and their activity was monitored following an 18 h incubation. Successful performance was achieved for both the ER-Ruby and the AR-BFP strains. The resulting fluorescence signals of the ER-Ruby strain are shown in Figure 3, and the corresponding dose-response curve in Figure 4. The respective results for the AR-BFP strain are displayed in Figure 5 and Figure 6.

### 3.2. Simultaneous Detection of Estrogenic and Androgenic Activities—Model Compounds

**Figure 7:**
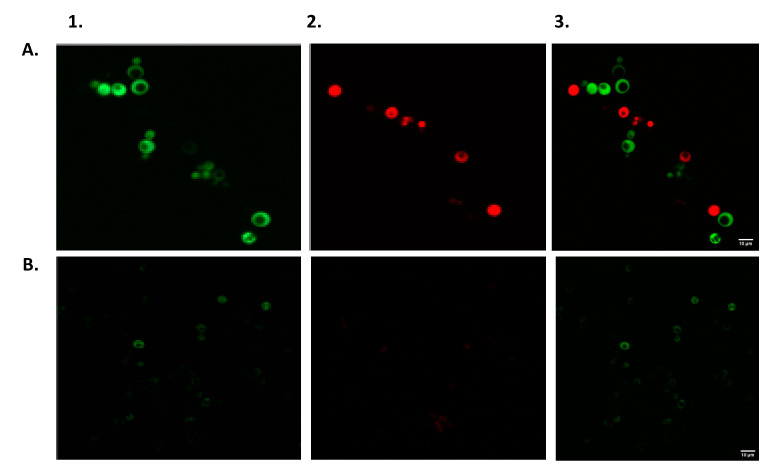
Simultaneous detection of estrogenic (E2) and androgenic (testosterone) model compounds by mixed ER-Ruby and AR-EGFP sensor strains in liquid culture. Images were obtained with a VF1200 confocal microscope (Olympus, Tokyo, Japan) with a 60 × 1.42 oil objective, following an 18 h incubation at 30 °C. Images were taken in a sequential mode using λ_ex_ = 488 nm and λ_em_ = 505–540 nm for the EGFP signal (column 1) and λ_ex_ = 561 nm and λ_em_ = 570λ620 nm for Ruby (column 2). Merged images are shown in column 3. Row (**A**)—a testosterone/E2 mixture, 250 ng/l each; Row (**B**)—no ligand.

To demonstrate the simultaneous detection of different EDC classes, we have tested the response of a 1:1 blend (v:v) of two of the newly constructed yeast fluorescent bioreporters, ER-Ruby (red fluorescence) and AR-EGFP (green fluorescence), to a E2/testosterone mixture. Exposure was first conducted in liquid culture; Figure 7 presents microscopic images obtained in the presence of different concentrations of the model inducers. Both strains have responded by a bright fluorescence of the respective newly synthesized reporter protein, with a larger number of fluorescent cells visible in the presence of the higher inducer dose.

**Figure 8:**
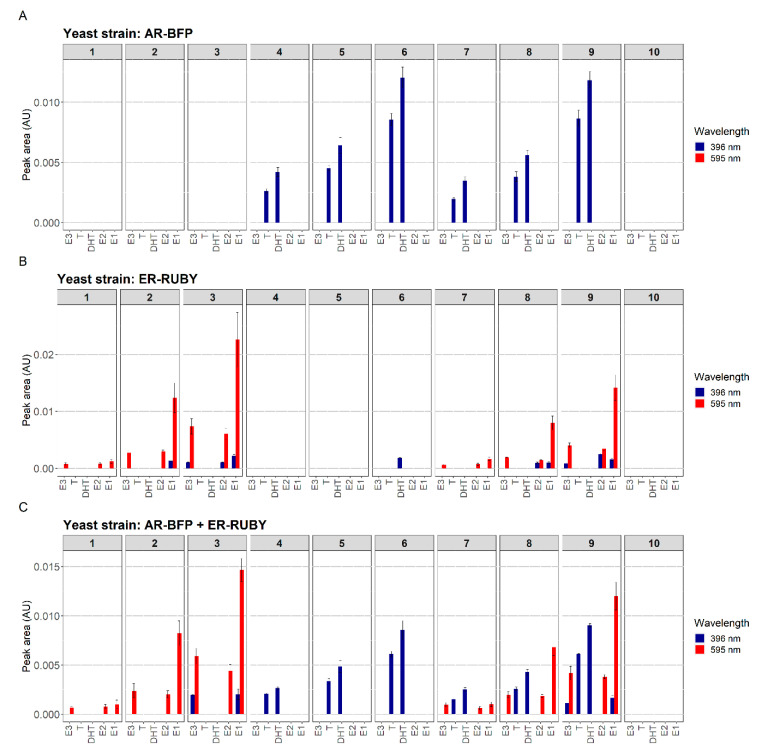
Multi-effect detection of chromatographically separated model compound mixtures by strain AR-BFP (**A**), strain ER-Ruby, (**B**) and both strains together (**C**). The HPTLC plates with the sprayed-on sensor cells were incubated for 18 h at 30 °C, following which fluorescence was scanned at λ = 396 nm and λ = 525 nm. Fluorescence intensities are presented in arbitrary units (AU). The estrogen mixture, separated on tracks 1–3 and 7–9, was composed of E1 (0.01 ng, 0.05 ng and 0.1 ng), E2 (0.005 ng, 0.01 ng and 0.02 ng), and E3 (0.5 ng, 1 ng and 2 ng). The androgen mixture, separated on tracks 4–6 and 7–9, was composed of testosterone (T; 0.5 ng, 1 ng and 5 ng) and DHT (0.5 ng, 1 ng and 5 ng). Ethanol served as blank on track 10. A two-step chromatographic development was performed, with 100% methanol for the first 20 mm, and an ethyl acetate/n-hexane 1:1 mixture for the next 70 mm.

Following the successful demonstration of the combined fluorescent detection of the two hormonal activities in liquid medium, we have investigated the possibility of combining the two assays also on the surface of an HPTLC plate. To characterize a possible crosstalk between the quantification of estrogenic and androgenic effects, two reporter strains (ER-Ruby and AR-BFP) were sprayed, either individually or in a 1:1 mixture, onto an HPTLC plate on which two blends of estrogenic and androgenic model compounds were chromatographically separated, either individually or in a mixture. The on-plate responses of the two reporter strains are presented in Figure 8. On all three plates, an estrogen mix consisting of E1, E2 and E3 and an androgenic mix consisting of testosterone and DHT were applied on tracks 1–3 and 4–6, respectively, while a combined mixture of both classes was applied on tracks 7–9. Activity detection was performed either with strain AR-BFP alone (top panel), ER-Ruby alone (middle panel) and with a mixture of the two reporter strains (bottom panel). The HPTLC plates were scanned at the wavelengths appropriate for the excitation of both fluorescent reporter proteins, and the fluorescence intensities are represented by the heights of the bars in Figure 8. These data were also used to calculate the LOD values for the different compounds, as presented in Table 2.

The blue fluorescence sensor strain AR-BFP detected the two androgenic model chemicals (Figure 8A), when on their own (tracks 4–6) as well as in the presence of the estrogenic mixture (tracks 7–9). Similarly, the red fluorescence sensor ER-Ruby displayed a clear response (Figure 8B) to the three estrogenic compounds, both in the absence (tracks 1–3) and the presence (tracks 7–9) of the androgenic substances. In all cases, the responses were dose-dependent. The intensity of the responses when both hormone classes were combined (Figure 8C) tended to be lower than when the mixtures were separated. This was more evident in the case of the ER-Ruby sensor (Figure 8B), which also displayed a minor response to the higher doses of the androgenic compounds. The reduced sensitivities in the combined presence of the two hormone classes are also evident from the higher LODs listed in Table 2.

### 3.3. Simultaneous Detection of Estrogenic and Androgenic Activities—Wastewater Samples

**Figure 9:**
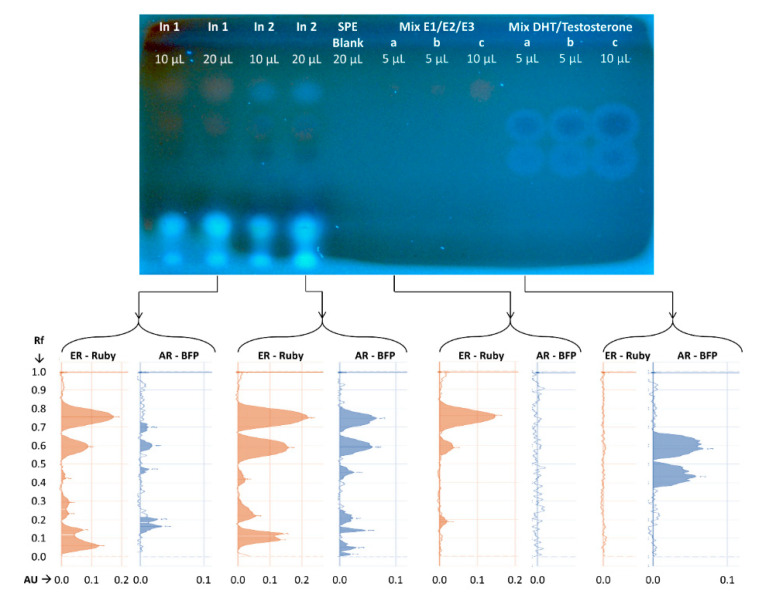
Simultaneous detection of androgenic and estrogenic effects in extracts of two wastewater treatment plants (WWTP) influent (In1, In2; 10 µl and 20 µl each) by a 1:1 mix of two yeast bioreporter strains, ER-Ruby and AR-BFP. An estrogen mix consisting of E1 (a: 0.1, b: 0.2 and c: 0.4 ng), E2 (0, 20 and 40 pg) and E3 (1, 2 and 4 ng), and an androgen mix of DHT (a: 10, b: 25 and c: 50 ng) and testosterone (10, 25 and 50 ng) were separated on the same plate. Ethanol served as blank on track 10. A two-step chromatographic development was performed, with 100% methanol for the first 20 mm, and an ethyl acetate/n-hexane 1:1 mixture for the next 70 mm. Top: plate image displaying the fluorescent signal using a TLC Visualizer 2 at λ_ex_ = 366 nm following an 18 h exposure at 30 °C. Signals were enhanced using the enhancement tool of the *visionCATS* software. Bottom: scans of the individual tracks for both ER-Ruby- and AR-BFP-fluorescence (TLC Scanner settings: Ruby: λ_ex_ = 525 nm, a 540 nm cutoff filter; BFP: λ_ex_ = 396 nm a 400 nm cutoff filter).

The robustness of the HPTLC combined bioassay using the newly developed fluorescent yeast sensor cells was further demonstrated by its application to wastewater samples. Concentrated influent samples from two municipal wastewater treatment plants (WWTPs) were separated by HPTLC, following which the plates were sprayed with a 1:1 mixture of two reporter strains, ER-Ruby and AR-BFP, and incubated for 18 h at 30 °C. An estrogenic mix consisting of E1, E2, and E3 and an androgenic mix consisting of DHT and testosterone were identically treated. Figure 9 displays an image of the plate (top) and the fluorescence scans of the individual lanes (bottom).

As is evident from the data in Figure 9, both influent samples contain components with potential estrogenic and androgenic activities. For example, the Ruby fluorescent signal, detected in both influent samples at Rf = 0.75, is similar in its chromatographic migration distance to that of E1, hinting at the presence of a molecule with an estrogenic activity chemically resembling E1. In the same location in sample In2 there is also an apparent androgenic activity, which is not shared by sample In1. Additional fluorescent signals were detected at Rf = 0.6 in sample In 2, and to a lesser extent also in In1 for both yeast bioreporter strains. These signals showed a similar migration behavior as the estrogenic model compound E2 and the androgenic model compound DHT. Clearly, however, while the fluorescent intensity of each active sample constituent on the HPTLC plate can be accurately quantified, a determination of its actual concentration cannot be performed before it is fully identified by analytical chemical means.

## 4. Discussion

Cell-based assays utilizing reporter gene technology have been widely promoted for environmental monitoring; in contrast to chemical analysis, they provide information about the biological effects of a tested sample, even if its exact composition is unknown. Furthermore, such assays can be tailored to detect chemicals with specific modes of action, such as an interference with cellular processes, including hormone receptor signaling [35,36].

Many assays for the detection of endocrine disrupting effects employ yeast cells as the cellular chassis, and rely on the expression of *LacZ* as the reporting element [25,26,37,38]. However, to measure the level of *LacZ* expression, the cell membrane must be disrupted, and an external substrate needs to be added to visualize the extent of gene expression induced by the target chemical(s). In contrast, fluorescent proteins provide a fast and accurate alternative [39,40] for signal detection even in living cells. Furthermore, as demonstrated in the present article, it allows the multi-parallel detection of several types of fluorescent proteins, differing in their optical attributes. Here we have described a set of *Saccharomyces cerevisiae* fluorescent protein-based bioreporters for the detection of two classes of endocrine disrupting chemicals in complex samples, in combination with sample separation by HPTLC, with LOD values similar or lower to those reported for other cellular assays (Table 2).

Probably the most relevant comparison of our results is to those described in the recently published reports of an *Arxula Adeninivorans*-based cell assay, employing three fluorescent bioreporter strains for the detection of estrogenic, androgenic, and progesterone activity, combined with HPTLC [19]. In that study, a clear peak with the fluorescent *Arxula adeninivorans* yeast strain G1212/YRC102-hER-DsRed, was detected for E2 > 0.0075 ng, without chromatographic separation. This result indicates a similar sensitivity compared to the strain generated in the current study, hER-Ruby, with a LOD of 0.008 ng, after chromatographic separation. The minimal detectable amount of DHT by the YRC102-hAR-GFP strain was 0.025 ng [19], lower than our strain hAR-BFP with a LOD < 0.5 (following chromatographic separation). However, a direct comparison of the performance characteristics of the bioreporter strains described by Chamas et al. [19] is not possible, since in the current study the cells were applied to the plate surface by spraying, compared to immersion of the developed plate in a suspension of the yeast bioreporters. The spray-on technology, as described herein, allows control over the thickness of the yeast layer as well as the plate’s moisture, producing clear and sharp bands [20]. Furthermore, as noted above, the LOD values reported here were determined following full chromatographic migration and separation.

A main objective of the current study was to allow a multi-parallel effect detection of both estrogenic and androgenic activities by complex samples and their components. This was achieved by combining sample separation by HPTLC with the concomitant application of two representative sensor strains; in these, red and blue fluorescence are induced in the presence of estrogenic or androgenic compounds, respectively. The general functionality of both reporters in parallel is demonstrated in Figure 8 and Figure 9. The former figure describes a systematic experiment addressing the possible crosstalk between the assays, in terms of false positive signals and effects on assay sensitivity. In the case of the AR-BFP strain, intensity of the signal caused by the androgenic model compounds remained virtually unchanged in the presence of the estrogenic model compounds. Furthermore, no false positive results were detected at 595 nm, indicating that the potential presence of estrogenic compounds is detected specifically by the ER-Ruby strain even in the presence of androgenic compounds. A possible crosstalk between androgenic compounds such as DHT and an activation of the estrogen receptor is discussed in literature either via a direct binding of DHT to the estrogen receptor [36] or due to a metabolic conversion of testosterone [41,42], but was not found in the present study.

Nevertheless, apparent false positive signals were detected at 396 nm as a strain ER-Ruby response, on its own, to estrogenic compounds. The increasing intensity of this signal correlates with the increasing signals at 595 nm in the presence of higher concentrations of the model compounds. Possibly, when the concentration of this fluorescent protein is sufficiently high, the low excitation of Ruby at 396 nm becomes visible. This artefact may be avoided by the introduction of a better-tuned filter system. The 595 nm signal intensity, indicating the presence of the Ruby protein, is lower when the estrogenic and androgenic model compounds are applied in a mixture. While actual signal intensities remain quite stable in the co-exposure (Figure 8), assay sensitivity is reduced due to an increased noise level leading to reduced S/N-values. While future refinement of the optical system is certainly desirable, the results presented in Figure 8 clearly demonstrate the possibility of a specific multi-parallel effect detection.

The specificity and robustness of the constructed yeast strains and the adaptability of the combined method for screening environmental samples is demonstrated by the characterization of influent samples from two different WWTPs. As in any chromatographic system, TLC separation of a complex sample into its components depends on adsorption properties of the compounds to the solid phase, as well as their solubility and migration distance with the mobile phase. Since estrogens and androgens share similar structures and physico-chemical properties, it is challenging to separate the two classes from each other with HPTLC [19]. The use of the multi-parallel effect detection is thus advantageous, as different effects may be detected even if the active compounds are not fully separated. This is clearly visible for the influent sample from the second WWTP (sample In 2). In this sample, two strong signals for estrogenicity and androgenicity at Rf-values of 0.75 and 0.61 are superimposed. The estrogenic bands at Rf 0.75 and 0.61 migrate similarly to E1 and E2, respectively; the androgenic compound Rf migrates similarly to DHT, while no candidate compound can be assigned to the androgenic signal at Rf = 0.75. Interestingly, no androgenic signals above a signal to noise ratio of 3 could be detected in the influent sample of WWTP 1 at these positions, underlining the specificity of the detection even in complex environmental samples.

## 5. Conclusions

We have presented a panel of sensitive, specific, and robust fluorescent bioreporter *S. cerevisiae* strains for the detection of compounds exerting estrogenic and androgenic effects in complex samples, and have demonstrated their efficacy in the analysis of both model compounds and multi-component wastewater samples. The use of fluorescent proteins as reporter elements obviates the requirement for cell lysis, substrate addition, and a second incubation step. The information emerging from such assays, when combined with thin layer chromatographic separation, can serve to restrict subsequent chemical analysis only to the small number of active fractions of the sample; this; in turn, will allow significant savings in time and costs, and a more focused and efficient risk assessment. To further broaden the applicability of the approach, it would be desirable to expand the panel of sensor strains for additional hormone classes, lower limits of detection, and optimize separation conditions for different classes of endocrine and endocrine-like compounds. Furthermore, as for other whole-cell based assays, the approach described above is limited to the detection of compounds that are at least partially permeable into the intracellular environment; enhancing cellular permeability to a broader spectrum of potential target molecules should therefore be another objective in the design of future EDC sensor strains.

## Figures and Tables

**Table 1:** *Saccharomyces cerevisiae* strains and plasmids used in this study.

Strain or Plasmid	Description	Source or Reference
***S. cerevisiae* parental strains**		
hER	MATa; leu2; his3; Human estrogen receptor gene in the chromosome.	[25]
hAR	BJ 1991 MATa; prb1-1122; pep4-3; leu2; trp1; ura3-52; GAL Human estrogen receptor gene in the chromosome.	[26]
**Parental plasmids**		
pFA6a-link-yomRuby2/yomTagBFP2/yoEGFP	containing Ruby, BFP, EGFP gene respectively	Addgene #44858 #44839#44836 respectively [23]
pUTK407	Contains the *luxA* and *luxB* genes expressed from the bidirectional promoters GPD and ADH1 separated with two EREs.	[21]
pUTK420	Contains the *luxA* and *luxB* genes expressed from the bidirectional promoters GPD and ADH1 separated with four AREs.	[22]
**ER and AR reporter plasmids**		
ER fluorescent reporter (FP)	pUTK407 in which *luxB* was substituted by EGFP/Ruby/BFP gene.	This study
AR fluorescent reporter (FP)	pUTK420 in which *luxB* was substituted by EGFP/Ruby/BFP gene.	This study
**Fluorescent sensor strains**		
hER-EGFP	Contains the EGFP gene contred by ADH1 promoter and the *luxA* gene expressed from GPD promoter, with two repeats of EREs.	This study
hER-Ruby	Contains the Ruby gene contred by ADH1 promoter and the *luxA* gene expressed from GPD promoter, with two repeats of EREs.	This study
hER-BFP	Contains the BFP gene contred by ADH1 promoter and the *luxA* gene expressed from GPD promoter, with two repeats of EREs.	This study
hAR-EGFP	Contains the EGFP gene contred by ADH1 promoter and the *luxA* gene expressed from GPD promoter, with two repeats of EREs.	This study
hAR-Ruby	Contains the Ruby gene contred by ADH1 promoter and the *luxA* gene expressed from GPD promoter, with two repeats of EREs.	This study
hAR-BFP	Contains the BFP gene contred by ADH1 promoter and the *luxA* gene expressed from GPD promoter, with two repeats of EREs.	This study

**Table 2:** Limit of detection (LOD), limit of quantification (LOQ) and Rf values calculated for the ER–Ruby and AR-BFP sensor strains in response to HPTLC-separated reference compounds.

		Single Strain		Both Strains		Rf Values
		Individual	Mix	Individual	Mix	
E3	Mean LOD (ng)	0.82	1.43	1.7	3.6	0.19
	SE (ng)	0.02	0.09	0.4	1.9	
E2	Mean LOD (ng)	0.0081	0.012	0.03	0.04	0.60
	SE (ng)	0.0009	0.001	0.02	0.02	
E1	Mean LOD (ng)	0.0120	0.016	0.05	0.06	0.76
	SE (ng)	0.0002	0.002	0.03	0.04	
	Mean LOQ (ng)	0.042	0.058			
	SE (ng)	0.003	0.008			
Testosterone	Mean LOD (ng)	0.3	0.70	0.8 (*)	0.9	0.46
	SE (ng)	0.2	0.04	0.2	0.2	
DHT	Mean LOD (ng)	<0.5	<0.5	0.3 (*)	0.4	0.61
	SE (ng)			0.1	0.1	

(*) Two replicates only.

## Supplementary Materials

The following are available online at https://www.mdpi.com/2079-6374/10/11/169/s1, Table S1: Oligonucleotide primer sequences used for the construction of the plasmids in this study.
