# Supplementary material for: Yeast-Based Fluorescent Sensors for the Simultaneous Detection of Estrogenic and Androgenic Compounds, Coupled with High-Performance Thin Layer Chromatography
Source: Biosensors (Basel). 2020 Nov 8;10(11):169. doi: 10.3390/bios10110169 (PMC7695312; doi:10.3390/bios10110169)
Supplement: Supplementary file 1 [file biosensors-10-00169-s001.pdf]

Supplementary

Yeast-Based Fluorescent Sensors for the Simultaneous Detection of Estrogenic and Androgenic Compounds, Coupled with High-Performance Thin Layer Chromatography

Liat Moscovici [1], Carolin Riegraf [2,3], Nidaa Abu-Rmailah [1], Hadas Atias [1], Dror Shakibai [1], Sebastian Buchinger [2], Georg Reifferscheid [2] and Shimshon Belkin [1,*]

1 Department of Plant and Environmental Sciences, Institute of Life Sciences, Hebrew University of Jerusalem, Jerusalem 91904, Israel; liatmosc@savion.huji.ac.il (L.M.); nidaan8@gmail.com (N.A.-R.); hadas.atias@mail.huji.ac.il (H.A.); drors1987@gmail.com (D.S.)

2 Department Biochemistry, Ecotoxicology, Federal Institute of Hydrology (BfG), Am Mainzer Tor 1, 56068 Koblenz, Germany; carolin.riegraf@gmail.com (C.R.); Buchinger@bafg.de (S.B.); reifferscheid@bafg.de (G.R.)

3 RWTH Aachen University, Department of Ecosystem Analysis, Worringerweg 1, D-52074 Aachen, Germany

* Correspondence: shimshon.belkin@mail.huji.ac.il; Tel.: +972-2-6584192

Table S1. Oligonucleotide primer sequences used for the construction of the plasmids in this study.

	Primer	Primer Sequence 5' to 3'
1.	ADH1_**SpeI**_EGFP_For	GGGGGG**TCTAGT**AAAGAATTAATTAACATGTCAAAA
2.	ADH1_**Kpn1**_EGFP_Rev	GCTC**GGTACC**AATCTAGGCGCGCCTTACTTGTATAATTCA
3.	ADH1_**SpeI**_BFP_For	TTTTTTCCCGGG**ACTAGT**AAAGAAATGAGTGAGTTGATTAAGGA
4.	ADH1_**EcoRI**_BFP_Rev	TTTTTT**GAATTC**GAGCTCGGTACCAATCTATTAGTTTAGTTTGTGTCCCA
5.	ADH1_**SpeI**_Ruby_For	GGTGGT**GAATTC**TTACTTATACAATTCATCCA
6.	ADH1_**EcoRI**_Ruby_Rev	GGTGGT**GAATTC**TTACTTATACAATTCATCCA
